# Breast Cancer Prognosis Using a Machine Learning Approach

**DOI:** 10.3390/cancers11030328

**Published:** 2019-03-07

**Authors:** Patrizia Ferroni, Fabio M. Zanzotto, Silvia Riondino, Noemi Scarpato, Fiorella Guadagni, Mario Roselli

**Affiliations:** 1 BioBIM (InterInstitutional Multidisciplinary Biobank), IRCCS San Raffaele Pisana, Via di Val Cannuta 247, 00166 Rome, Italy; silvia.riondino@sanraffaele.it (S.R.); fiorella.guadagni@sanraffaele.it (F.G.); 2 Department of Human Sciences & Quality of Life Promotion, San Raffaele Roma Open University, Via di Val Cannuta 247, 00166 Rome, Italy; noemi.scarpato@unisanraffaele.gov.it; 3 Department of Enterprise Engineering, University of Rome “Tor Vergata”, Viale Oxford 81, 00133 Rome, Italy; fabio.massimo.zanzotto@uniroma2.it; 4 Department of Systems Medicine, Medical Oncology, Tor Vergata Clinical Center, University of Rome “Tor Vergata”, Viale Oxford 81, 00133 Rome, Italy; mario.roselli@uniroma2.it

**Keywords:** breast cancer prognosis, artificial intelligence, machine learning, decision support systems

## Abstract

Machine learning (ML) has been recently introduced to develop prognostic classification models that can be used to predict outcomes in individual cancer patients. Here, we report the significance of an ML-based decision support system (DSS), combined with random optimization (RO), to extract prognostic information from routinely collected demographic, clinical and biochemical data of breast cancer (BC) patients. A DSS model was developed in a training set (*n* = 318), whose performance analysis in the testing set (*n* = 136) resulted in a C-index for progression-free survival of 0.84, with an accuracy of 86%. Furthermore, the model was capable of stratifying the testing set into two groups of patients with low- or high-risk of progression with a hazard ratio (HR) of 10.9 (*p* < 0.0001). Validation in multicenter prospective studies and appropriate management of privacy issues in relation to digital electronic health records (EHR) data are presently needed. Nonetheless, we may conclude that the implementation of ML algorithms and RO models into EHR data might help to achieve prognostic information, and has the potential to revolutionize the practice of personalized medicine.

## 1. Introduction

The breast cancer (BC) death rate has declined steadily over the past two decades, progress that can be attributed to the deployment of innovative management pathways, from early detection to treatment. Nevertheless, BC still represents the leading cause of cancer death among females worldwide [1]. Accordingly, BC survivability prediction represents a challenging task that could strongly benefit from the development of personalized predictive models. In this context, contemporary oncology has witnessed a growing interest in digital technologies, whose integration with big healthcare data has raised new hopes for personalized medicine.

Artificial intelligence (AI) and machine learning (ML) have been used to diagnose and classify cancer for nearly 20 years, but only a few studies have investigated their relevance in cancer prognosis [2]. In particular, ML or semi-supervised learning techniques have been recently applied to develop models for BC progression and survivability. Most of them, however, were built on datasets from the SEER (Surveillance Program, Epidemiology, and End Results), not including important prognostic parameters such as the St. Gallen criteria (hormones receptor status, HER2/Neu expression or Ki67 proliferation index) [3,4,5], while other studies were performed on hybrid models containing microarray data [6] or on mammographic images [7,8]. Lately, an unsupervised ML approach that can admit any number of prognostic factors, was used to build prognostic systems for cancer patients [9]. Also, in this case, the SEER dataset used did not include information on HER2/Neu expression, whose prognostic significance has been emphasized in the 8th edition TNM staging system for BC [10]. Thus, the unmet need to develop prognostic classification models that embody the newest AI technologies and can be used to predict outcomes in individual cancer patients for personalized patient care has been highlighted [11].

In this context, we have recently demonstrated the potential of a semi-explainable decision support system (DSS), based on multiple kernel learning (MKL) [12], that can be adapted to different medical problems [13,14] and gives the possibility to inspect the learned model. The model combines a support vector machine (SVM) [15] algorithm and random optimization (RO) [16]. Hence, it can offer an explanation on how routinely collected demographic, clinical and biochemical data are important in predictions. This MKL model, originally developed for cancer-associated thrombosis risk assessment [13], has been here adapted to estimate the risk of disease progression in an oncology setting of BC patients. To achieve this objective, a proof-of-concept study was specifically designed to assess whether a customized MKL-based DSS could be a useful prognostic tool in the clinical management of BC patients.

## 2. Results

A set of predictors (named ML-RO) was identified using a 3-fold cross-validation technique on a training set (*n* = 318). A testing set (*n* = 136) was used to compute the final performance of risk predictors. To devise the DSS, we selected ML-RO-4 as the best performing out of a range of ten runs, in terms of the area under the curve (AUC), on the training set (Table 1).

As shown in Table 1, most predictors had a receiver operating characteristic (ROC) curve with an AUC ≥0.75 (the threshold generally accepted as clinically useful) [17]. Among these, ML-RO-0 was further selected as it provided a major relative importance to the group of features linked to glucose metabolism (Group 5) (Table 2), which is currently considered an important contributor to BC progression [18,19], at the point that metformin—an anti-diabetic drug with insulin-lowering effects—has been proposed in combination with chemotherapy [20,21] and is currently being considered vs. placebo in a phase-III randomized trial in early stage BC (ClinicalTrials.gov Identifier: NCT01101438).

When both predictors were incorporated into a DSS model for BC progression, their combined use (both positive, either positive, both negative) in the testing set translated in a c-statistic = 0.84 (95% CI: 0.76–0.90). The level with the best Youden index at ROC analysis (>1, i.e., risk estimate achieved by both predictors, according to voting on the positive class) was then selected as the cutoff value for further evaluation of the combined DSS. A comparison of the analytical performance of the trained models and derived DSS on the testing set is reported in Table 3. 

**Figure 1:**
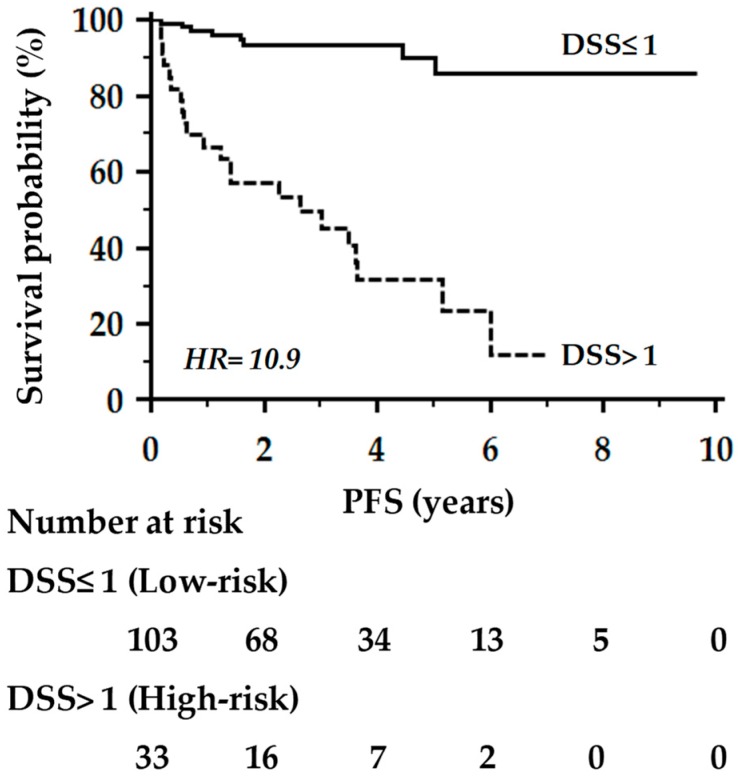
Kaplan–Meier curves of progression-free survival (PFS) of the 136 BC women included in the testing set. Comparison between patients at high (>1) or low-risk (≤1) of progression by the combined decision support system (DSS) model.

At a criterion >1, the DSS model was capable of stratifying primary BC patients into two groups with a low- or high-risk of progression, either in the training (*n* = 279; log-rank = 3.23, *p* = 0.001) or in the testing set (*n* = 118; log-rank = 3.42, *p* < 0.001). Figure 1 reports the Kaplan–Meier curves of progression-free survival (PFS) in the 136 BC women included in the testing set and followed-up for a mean time of 3.5 years (ranging from 0.3–9.7 years). As shown, patients estimated at high risk (>1) of progression by the combined DSS model had a 5-year progression-free survival probability significantly lower than that observed in BC patients estimated at low-risk (≤1) (26% vs. 85%, respectively; log-rank = 6.82, *p* < 0.0001).

## 3. Discussion

Treatment decisions are particularly challenging in early-stage BC patients with conflicting prognostic features, especially node-negative ones, in which the question of whether to pursue an adjuvant treatment with chemotherapy or endocrine therapies is still unclear. Putative biomarkers, so far, have not demonstrated sufficient predictive ability to be clinically useful. Ki67 itself lacks reproducibility and its use, if not part of an AI model, has been largely re-dimensioned [22].

Identification of predictive tools of tumor responsiveness, risk of recurrence, and mortality, providing the possibility to avoid unnecessary toxicities are thus very appealing. As reported above, ML has started to take hold across the oncology community to develop prognostic classifications models of BC progression and survivability [9]. In this regard, the possibility to perform an automated survival prediction in metastatic cancer patients using high-dimensional electronic health records (EHR) data has been recently highlighted [23]. By using an ML approach on EHR-derived predictor variables (clustered into categories), Gensheimer et al., in fact, devised an AI system, with a better c-statistic than previously reported prognostic models, which could be deployed in a DSS to help improve quality of care in the metastatic setting [23]. More recently, four major nonlinear ML methods (integrating multiple clinicopathological features and genomic data) were used to compare survival predictions in a large cohort of BC patients [24]. Although no model significantly outperformed others, the Nottingham Prognostic Index, age, tumor stage and size, ER/PR/HER2 and breast surgery status strongly influenced survival across repeated runs and models, while the gene expression cluster was a moderately influential factor [24].

The results here reported confirm and extend the findings by Zhao et al., as the use of an SVM has proven effective in devising an AI-based DSS for the prognostic assessment of non-metastatic BC patients. In particular, the combined use of ML and RO techniques, allowed the construction of a set of prognostic discriminators from routinely collected clinicopathological features and biochemical data of BC patients, which showed a better performance than the predictors developed by Zhao et al. (c-statistic 0.82 vs. 0.66 and an accuracy of 86% vs. 73%, respectively) [24]. In our opinion, this combined approach might hold potential for improving model precision through weighting the relative importance of attributes. Moreover, with respect to models based on neural networks [7], the combination of ML and RO techniques offers a model that can be learned with small datasets and that is more interpretable, as were Bayesian networks applied to BC [25]. Furthermore, the devised DSS included a number of prognostic and metabolic parameters, not previously analyzed, that could be easily extracted by EHR, meaning that ML may add significant and sustained benefits to personalized medicine at no additional cost to the health system. 

Of course, there are limitations to acknowledge. First, the study was mono-institutional. Second, the sample size was relatively small, which may have lowered the power of ML. Nonetheless, we believe that implementation of ML algorithms and RO models into high-dimensional EHR data might help to achieve prognostic information, and has the potential to revolutionize the practice of personalized medicine. 

## 4. Patients and Methods

Starting from January 2007, the PTV Bio.Ca.Re. (Policlinico Tor Vergata Biospecimen Cancer Repository) and the SR-BioBIM (Interinstitutional Multidisciplinary Biobank, IRCCS San Raffaele Pisana, Rome, Italy) are actively involved in the recruitment of ambulatory patients with primary or metastatic cancer, who are prospectively followed under the appropriate institutional ethics approval, as part of a Clinical Database and Biobank project. Among these, a cohort of 454 consecutive BC patients in whom prognostic and pre-treatment biochemical factors were available, were selected for the present analysis. The study was performed in accordance with the principles embodied in the Declaration of Helsinki. All patients gave written informed consent, previously approved by our Institutional Ethics Committee (ISR/DMLBA/405, 15 November 2006). BC was pathologically staged according to the latest prognostic TNM staging system [8]. Three hundred and ninety-seven women (87%) had primary BC and underwent radical surgery followed by radiation and/or adjuvant treatment as per current guidelines. The remaining 57 (13%) patients presented with metastatic disease. Prognostic routinely-collected factors such as BC stage, menopausal status, pathological grading as well as the St. Gallen criteria (e.g., estrogen and progesterone receptors, HER2/neu expression and the proliferation index Ki67) were available for each patient. In particular, grading was assessed according to the Nottingham grading system (Elston–Ellis modification of the Scarff–Bloom–Richardson grading system) for BC [8]. The immunohistochemical analyses were performed on formalin-fixed, paraffin-embedded tumor sections for hormone receptor presence [26], HER2/neu expression [27] and proliferation index (Ki67) [28]. HER2/neu positivity was defined according to the American Society of Clinical Oncology-College of American Pathologists (ASCO-CAP) guidelines as an immunohistochemical staining of 3+ or 2+ with evidence of gene amplification by fluorescence in situ hybridization (FISH) [27]. The Ki67 proliferative index in surgical specimens was assigned by the pathologist based on the percentage of positivity on at least 500 neoplastic cells counted in the peripheral area of the nodule. A cut–off value of ≥20% was used in all association analyses, according to the recommendations of the St. Gallen International Expert Consensus on the primary therapy of early BC 2013 [28].

Furthermore, given the increasing awareness that metabolic features might represent an important contributor to BC progression, Type 2 diabetes, glycemic parameters and the body mass index (BMI) were introduced in the model [18,19]. Routine biochemical analyses were performed on fresh blood samples taken in the morning after an overnight fast at the time of enrolment and prior to any treatment (surgery, adjuvant, either chemotherapy or endocrine, or metastatic). The demographic and clinical characteristics of the recruited population are summarized in Table 4.

The machine learning used for the primary analysis was run using the kernel-based learning platform (KeLP) [29], as previously reported [13]. Multiple kernel learning (MKL), based on support vector machines (SVM) and random optimization (RO) models, were used to produce prognostic discriminators (referred as machine-learning (ML)-RO) yielding the best classification performance over a training (3-fold cross-validation) and testing set. The training set consisted of 318 BC patients (70% of the dataset); the remaining 136 patients were allocated to the testing set (30% of the cases). No significant difference was observed for demographic, clinical and biochemical characteristics between the training and testing set (Table 4). The numerical attributes were analyzed as continuous values. Missing clinical attribute values were treated according to the predictive value imputation (PVI) method by replacing missing values with the average of the attribute observed in the training set. The variables were clustered into five groups according to clinical significance. A detailed list of all the features applied to construct the predictor is reported in Table 5. RO was used to devise their relative weights in the final prediction. In RO, relative weights are initialized with a random number and estimated by maximizing performance in the 3-fold cross-validation. These weights can be used to interpret the importance of the groups of features within the model. Thus, the final DSS is interpretable. 

Statistical analysis

The receiver operating characteristic (ROC) curve and univariate Cox proportional hazards analyses were performed by MedCalc Statistical Software version 13.1.2 (MedCalc Software bvba, Ostend, Belgium). The area under the curve (AUC) was calculated on a three-level risk: 2 (if both predictors estimated the risk), 1 (if only one predictor estimated the risk) or 0 (if both predictors did not estimate the risk) to investigate whether the combined DSS could distinguish between recurrent and non-recurrent patients. The level with the best Youden index (>1, i.e., risk estimate achieved by both predictors) was selected as the cutoff value for the combined DSS. Bayesian analysis was performed, and positive (+LR) and negative (−LR) likelihood ratios were used to estimate the probability of BC progression. The survival curves were calculated by the Kaplan–Meier and log-rank methods using computer software packages (MedCalc Software bvba, Ostend, Belgium and Statistica 8.0, StatSoft Inc., Tulsa, OK, USA). The PFS represented the study endpoint and was calculated from the date of enrollment until disease progression. The patients who had no disease progression were censored at the time of the last follow-up. For administrative censoring, the follow-up ended on 31 December, 2017. All tests were two-tailed and only *p*-values lower than 0.05 were regarded as statistically significant.

## 5. Conclusions

ML has recently started to take hold across the oncology community to develop prognostic classifications models of cancer progression and survivability. In our opinion, a combined approach of ML algorithms and RO models might hold potential for improving model precision through weighting the relative importance of attributes. In line with the actual trend, in fact, the proposed model seeks not only decision, but also interpretability of the model itself, which, together with the use of a real-world BC dataset, represents the novel aspect of our research. Validation in multicenter prospective studies and appropriate management of privacy issues in relation to digital EHR data are required before making any ML approach into the clinical practice available.

## Figures and Tables

**Table 1:** Analytical performance of machine learning with random optimization in the training set.

ML Predictor	AUC (SE)	95% CI	Sensitivity (95% CI)	Specificity (95% CI)	+LR	−LR
**ML-RO-4**	0.778 (0.0290)	0.728–0.822	67.1 (55.4–77.5)	88.4 (83.7–92.2)	5.80	0.37
**ML-RO-1**	0.769 (0.0293)	0.719–0.814	65.8 (54.0–76.3)	88.0 (83.2–91.8)	5.49	0.39
**ML-RO-7**	0.767 (0.0293)	0.717–0.813	67.1 (55.4–77.5)	86.4 (81.4–90.4)	4.92	0.38
**ML-RO-3**	0.759 (0.0296)	0.708–0.805	65.8 (54.0–76.3)	86.0 (80.9–90.1)	4.68	0.40
**ML-RO-6**	0.759 (0.0296)	0.708–0.805	65.8 (54.0–76.3)	86.0 (80.9–90.1)	4.68	0.40
**ML-RO-8**	0.755 (0.0297)	0.703–0.801	65.8 (54.0–76.3)	85.1 (80.0–89.4)	4.42	0.40
**ML-RO-0**	0.753 (0.0297)	0.701–0.799	65.8 (54.0–76.3)	84.7 (79.5–89.0)	4.30	0.40
**ML-RO-2**	0.748 (0.0299)	0.697–0.795	64.5 (52.7–75.1)	85.1 (80.0–89.4)	4.33	0.42
**ML-RO-9**	0.739 (0.0302)	0.687–0.786	61.8 (50.0–72.8)	86.0 (80.9–90.1)	4.40	0.44
**ML-RO-5**	0.722 (0.0306)	0.669–0.770	59.2 (47.3–70.4)	85.1 (80.0–89.4)	3.98	0.48

AUC: Area under the curve; CI: Confidence interval; LR: Likelihood ratio; ML: Machine learning; RO: Random optimization.

**Table 2:** Weights of attribute groups in the training set.

Method	Group					Sum of the Weights	Normalized Group Weights				
	1	2	3	4	5		1	2	3	4	5
**ML+RO-4**	0.41890	1.04551	0.60311	0.33909	0.58969	2.996321	0.13980	0.34893	0.20128	0.11316	0.19680
**ML+RO-0**	0.77299	1.86062	1.39445	0.90456	1.00740	5.940053	0.13013	0.31323	0.23475	0.15228	0.16959
**ML+RO-6**	0.42756	0.91373	1.16514	0.39297	0.58755	3.486968	0.12261	0.26204	0.33414	0.11269	0.16849
**ML+RO-8**	0.44878	1.28224	0.63075	0.44350	0.53398	3.339267	0.13439	0.38399	0.18888	0.13281	0.15991
**ML+RO-1**	0.46149	1.17742	0.55782	0.34141	0.47660	3.014770	0.15307	0.39055	0.18503	0.11324	0.15809
**ML+RO-7**	0.54682	1.40025	0.79264	0.59119	0.61023	3.941154	0.13874	0.35529	0.20112	0.15000	0.15483
**ML+RO-3**	0.64274	1.13249	0.36078	0.39482	0.45241	2.983255	0.21545	0.37961	0.12093	0.13234	0.15165

Data are absolute numbers for group weights. ML: Machine Learning; RO: Random Optimization.

**Table 3:** Analytical performance of machine learning with random optimization in the testing set.

Performance Parameter	ML-RO-0	ML-RO-4	DSS Model ^a^
**F-measure** ^b^	0.696	0.677	0.698
**Accuracy**	0.853	0.838	0.860
**Area under the curve (AUC)**	0.822	0.813	0.815
**(+)LR (95% CI)**	9.1 (4.3–20.8)	8.5 (3.9–19.6)	8.6 (4.2–18.0)
**(−)LR (95% CI)**	0.4 (0.3–0.6)	0.4 (0.3–0.6)	0.4 (0.2–0.5)
**HR (95% CI)**	10.7 (4.6–24.8)	10.3 (4.5–23.7)	10.9 (4.5–26.6)

LR: Likelihood ratio; C.I.: Confidence interval; HR: Hazard ratio; ^a^ Analytical performance was evaluated after categorization 0/1 based on risk estimate achieved by both predictors; ^b^ F-measure represents a harmonic mean of precision [(P) positive predictive value in machine learning] and recall [(R) sensitivity in machine learning] and is calculated as: 2PR/(P+R).

**Table 4:** Clinical-pathological characteristics of breast cancer (BC) patients. Comparison between training and testing set.

Clinical-Pathological Characteristics	Training Set (*n* = 318)	Testing Set (*n* = 136)
Age (years), Mean ± SD	56 ± 13	57 ± 12
Menopausal status, N (%)		
Pre	141 (44)	51 (38)
Post	177 (56)	85 (63)
Body Mass Index, Mean ± SD	25.2 ± 4.5	25.7 ± 5.2
Histological diagnosis, N (%)		
Ductal	263 (83)	121 (89)
Lobular	37 (12)	9 (7)
Others	18 (5)	6 (4)
Molecular Type ^a^, N (%)		
Triple-negative	39 (12)	17 (12)
Luminal-like A	97 (31)	37 (27)
Luminal-like B	172 (54)	77 (57)
HER2 pos	10 (3)	5 (4)
Grading, N (%) ^b^		
1	20 (7)	15 (13)
2	108 (39)	45 (38)
3	151 (54)	58 (49)
Tumor, N (%) ^b^		
T1	141 (50)	59 (50)
T2	91 (33)	42 (36)
T3	28 (10)	5 (4)
T4	19 (7)	12 (10)
Node, N (%) ^b^		
N0	134 (48)	54 (46)
N+	145 (52)	64 (54)
Prognostic stage, N (%)		
I	177 (56)	70 (50)
II	53 (17)	20 (15)
III	45 (14)	26 (19)
IV	4 (1)	2 (1)
Metastatic	39 (12)	18 (13)
Receptor status, N (%) ^c^		
ER+/PR+	235 (74)	94 (69)
ER+/PR−	29 (9)	19 (14)
ER-/PR+	5 (2)	1 (1)
ER-/PR−	49 (15)	22 (16)
HER2/neu+, N (%) ^c^	66 (21)	34 (25)
Ki67 proliferation index ≥20%, N (%) ^c^	204 (67)	93 (71)
Type 2 Diabetes, N (%)	39 (12)	11 (8%)
Glucose metabolic asset ^d^		
Fasting blood glucose (mg/dl), Mean ± SD	105 ± 31	102 ± 32
Fasting insulin (µIU/ml), Median (IQR)	11.9 (6.4–27.0)	10.6 (5.6–19.6)
HbA_1c_ (%), Mean ± SD	5.8 ± 0.8	5.8 ± 0.7
HOMA Index, Mean ± SD	3.0 (1.4–8.3)	2.9 (1.2–6.3)
Follow-up (years)		
Mean (range)	3.4 (0.29–10.5)	3.5 (0.26–9.65)

^a^ According to St. Gallen Consensus Conference. ^b^ Evaluated at time of diagnosis. ^c^ Evaluated in a population of 397 primary breast cancer patients. ^d^ Evaluated at time of enrollment and prior to any treatment. ER/PR: estrogen/progesterone receptors; HER2: Human epidermal growth factor receptor 2; IQR: Interquartile range; HbA_1c_: Glycosylated hemoglobin; HOMA Index: Homeostasis model assessment index.

**Table 5:** Features included in the model.

Patient-Related	Tumor-Related	Biochemical
**Group 1:** Age Menopausal status Body Mass Index	**Group 2:** Molecular type Histological diagnosis Grading TNM stage	**Group 4:** Total Bilirubin Creatinine
		**Group 5:** Fasting glycemia Fasting insulinemia Glycosylated hemoglobin HOMA index (insulin resistance) Type 2 diabetes
	**Group 3:** Estrogen receptors Progesterone receptors HER2/NEU Ki67 proliferation index	
